# Antibiotic Conditioning Shapes Pseudosterile Mouse Models by Deleting Colonic Microbes Rather than Small Intestinal Microbes

**DOI:** 10.1128/spectrum.00814-23

**Published:** 2023-04-19

**Authors:** Qingxue Chen, Xinming Ma, Zhishuang Xing, Xin Zhao, Hang Zu, Zengwang Guo, Bailiang Li

**Affiliations:** a Key Laboratory of Dairy Science, Ministry of Education, Northeast Agricultural University, Harbin, China; b Food College, Northeast Agricultural University, Harbin, China; c CABIO Biotech (Wuhan) Co., Ltd., Wuhan, China; d Heilongjiang Ubayt Dairy Group Co., Ltd., Qiqihaer, China; Lerner Research Institute

**Keywords:** antibiotics, deletion of intestinal microorganisms, colon, mouse model

## Abstract

A simple model of alternative microbiota in the developing intestinal environment has been highly desirable for the study of health and disease in the gut. The pattern of antibiotic depletion of natural gut microbes is necessary for this model. However, the effects and loci of antibiotic deletion of gut microbes remain unclear. In this study, a mixture of three proven broad-spectrum antibiotics was selected to study their effects on microbial deletions in the jejunum, ileum, and colon of mice. The 16S rRNA sequencing results showed that antibiotics significantly reduced colonic microbial diversity, with limited effects on the jejunum and ileum. At the level of microbial genera, only 93.38% of *Burkholderia-Caballeronia-Paraburkholderia* and 5.89% of *Enterorhabdus* were present in the colon after antibiotic treatment. However, such changes were not observed in the microbial composition of the jejunum and ileum. Our results suggest that the antibiotics depleted intestinal microorganisms by acting primarily in the colon and not in the small intestine (jejunum and ileum).

**IMPORTANCE** Many studies have applied antibiotics to delete intestinal microbes to shape pseudosterile mouse models and further used for fecal microbial transplantation. However, few studies have explored the spatial location of antibiotic action in the intestine. This study shows that the selected antibiotics effectively deleted microbiota in the colon of mice, with limited effects on microbes in the jejunum and ileum. Our study provides guidance for the application of a mouse model of antibiotic deletion of intestinal microbes.

## INTRODUCTION

The gut microbiota in the human body, known as another organ of the human body, has been one of the most interesting research focuses in the fields of microbiology, medicine, and genetics in recent years (1, 2). This is because the gut microbiota plays an important role in the health of the host, assisting in the decomposition of food, resisting intestinal pathogenic bacterial infection, and improving the body’s immunity (3, 4). Recent studies have found that the gut microbiota is inextricably linked with host acute (inflammation, allergy, and cancer) (5) or chronic diseases (obesity and autism) (6). Animal model experiments are often required for many studies of the gut microbiota. Germfree mice, in traditional thinking, are considered to be the best animal model for studying the gut microbiota. However, germfree animals are limited by a variety of factors and cannot meet the needs of all research. Therefore, the use of antibiotics to deplete the gut microbiota as an alternative to sterile mice was proposed (7, 8).

The most favorable aspect of germfree mice receiving exogenous gut microbiota is the lack of competition with resident microbes to colonize the intestine. However, the challenges of harsh feeding conditions or sterility on the health of the mice themselves seem to limit the application of germfree mice. The remaining challenges of germfree mice can be summarized as follows: (i) the cost of maintaining germfree mouse population acquisition and persistence is expensive (8); (ii) the absence of the microbiota may lead to persistent negative immune and metabolic consequences in germfree mice (9, 10); and (iii) many genotypic mouse models do not have a germfree option (7). To overcome these limitations, some researchers attempted to achieve a nearly sterile state by selectively depleting intestinal microbes using antibiotics; mice obtained in this form are known as pseudosterile mice (7, 8, 11). The pseudosterile mice effectively circumvent the limitations of the sterile mouse model (12). In addition, the pseudosterile mouse model can be adapted to the tolerance of target microorganisms to different antibiotics, resulting in mice with selective microbial depletion (12, 13). Given the diversity of intestinal microorganisms, the limitations of the range of action of antibiotics on microorganisms, and other factors, the composition, form of administration, and dose of antibiotics may be critical in determining whether pseudosterile mice will meet expectations. One study protocol chose an antibiotic (ciprofloxacin) to be administered continuously for 4 days, but the results were not significant (14). The use of a wider range of antibiotics was proved to be effective in many studies, for example, a mixture of two antibiotics (vancomycin and imipenem) administered for 3 days (15), a mixture of four antibiotics (neomycin, metronidazole, vancomycin, and ampicillin) administered for 2 days (16), and a mixture of five antibiotics (ampicillin, vancomycin, neomycin, metronidazole, and amphotericin B) for 17 days (8).

The pseudosterile mouse model of antibiotic-depleted gut microbes is widely used because of its unique advantages. However, whether the antibiotic acts on the entire intestinal segment or on some specific locations is unclear. Therefore, we chose a proven and widely used antibiotic protocol to construct pseudosterile mice (7, 17). Notably, previous researchers compared them to germfree mice and elucidated that their use for further germfree transplantation is not inferior to that of germfree mice (7). Using genomic sequencing techniques, we examined the changes in microbial diversity and composition patterns in the jejunum, ileum, and colon following antibiotic administration. The location of antibiotic action in the intestine and the effect of antibiotic interference with microorganisms in different intestinal segments were analyzed. Based on this study, we attempted to provide some insights into the precise application of a pseudosterile model of antibiotic-depleted gut microbes.

## RESULTS AND DISCUSSION

### Antibiotics had a sufficient effect only on the colon.

**FIG 1:**
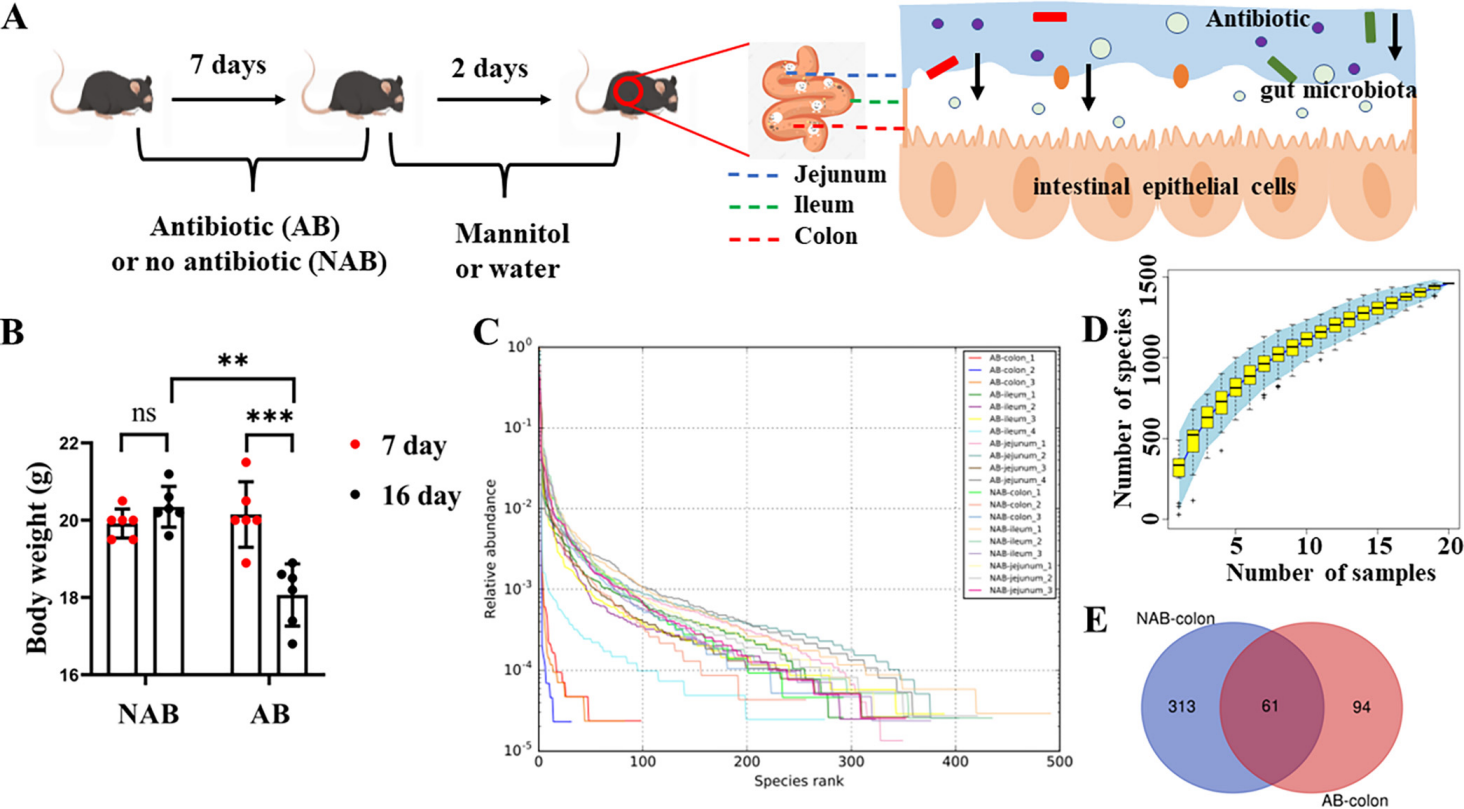
Experimental design of antibiotics to delete intestinal microorganisms in mice. (A) Animal experimental design and sample acquisition. (B) Body weight changes in mice (*n* = 6). (C) The rank curve based on OTUs. (D) Species accumulation curves. (E) Venn diagram of the number of OTUs in the colon. **, *P *< 0.01; ***, *P *< 0.001; ns, not significant.

Antibiotic-conditioned mice well depleted the number and composition of gut microbiota (11). However, whether these responses were directed at the entire gut or specific parts of the gut was not clear. We placed mice in antibiotic supplemented (AB) and no-antibiotic (NAB) drinking water to analyze the main battlefield of antibiotics in the antibiotic-depleted gut microbial mode. After 7 days of both treatments, the mice in the AB group were given 2 days of mannitol to empty the intestine, while the mice in the NAB group were given drinking water as a control (Fig. 1A). Obviously, the mice lost weight dramatically after receiving the antibiotics. The body weight of the mice in the AB group was significantly lower (*P* < 0.001) on the 16th day than on the 7th day, whereas the body weight of the mice in the NAB group had no significant change (*P* > 0.05) (Fig. 1B). Antibiotic exposures aimed at depleting gut microbes are often accompanied by a reduction in body weight in mice (18). This is inconsistent with the fact that antibiotic exposure early in life is associated with youth or long-term obesity(19, 20). In the case of weight gain due to early antibiotic exposure, the antibiotics increased the abundance of the obesity-associated microorganism Streptococcus, which influenced obesogenesis (20). However, the abundance of most microbes in the gut, including the obesity-associated bacterium Streptococcus, was dramatically reduced in this study and other studies aimed at depleting gut microbes. These changes may be a key factor in the reduction of body weight in mice. The composition of gut microbiota in the jejunum, ileum, and colon of mice was subsequently examined.

The similar sequences of mouse intestinal genes were clustered into operational taxonomic units (OTUs), and the homology of these similar gene sequences was more than 97% (21). The OTUs are the initial means of characterizing species diversity and richness. The rank curve based on OTUs is a form of presenting the species diversity in samples, which can explain both the richness and uniformity of the species contained in the sample. The distance of the rank curve on the horizontal axis represents the degree of species richness, whereas the rate of decline on the vertical axis represents the degree of species evenness (22). Figure 1C presents the rank curves of each sample in the six groups. For the jejunum and ileum samples, the rank curves in the AB and NAB groups were similar, which means longer distances and slower decreases. Notably, the rank curves of colon samples from mice in the AB group were significantly different from those in the NAB group, and the distance in the AB group was the shortest, while the curves showed a rapidly decreasing trend. This indicated that the jejunum microbiota diversity decreased after antibiotic treatment, presenting certain dominant bacteria as almost the entire composition of gut microbiota. In contrast, this phenomenon was not found in the jejunum and ileum. Correspondingly, the number of OTUs in the colon samples of the AB group was also significantly lower (*P* < 0.05) than that in the NAB group (Fig. 1E). The species accumulation curves reflect the rate of new OTU emergence under continuous sampling (22). As the sample size increased, the species accumulation curves gradually flattened out (Fig. 1D), indicating that the samples for analysis were sufficient.

### Marked reduction of microbiota diversity in the colon.

**FIG 2:**
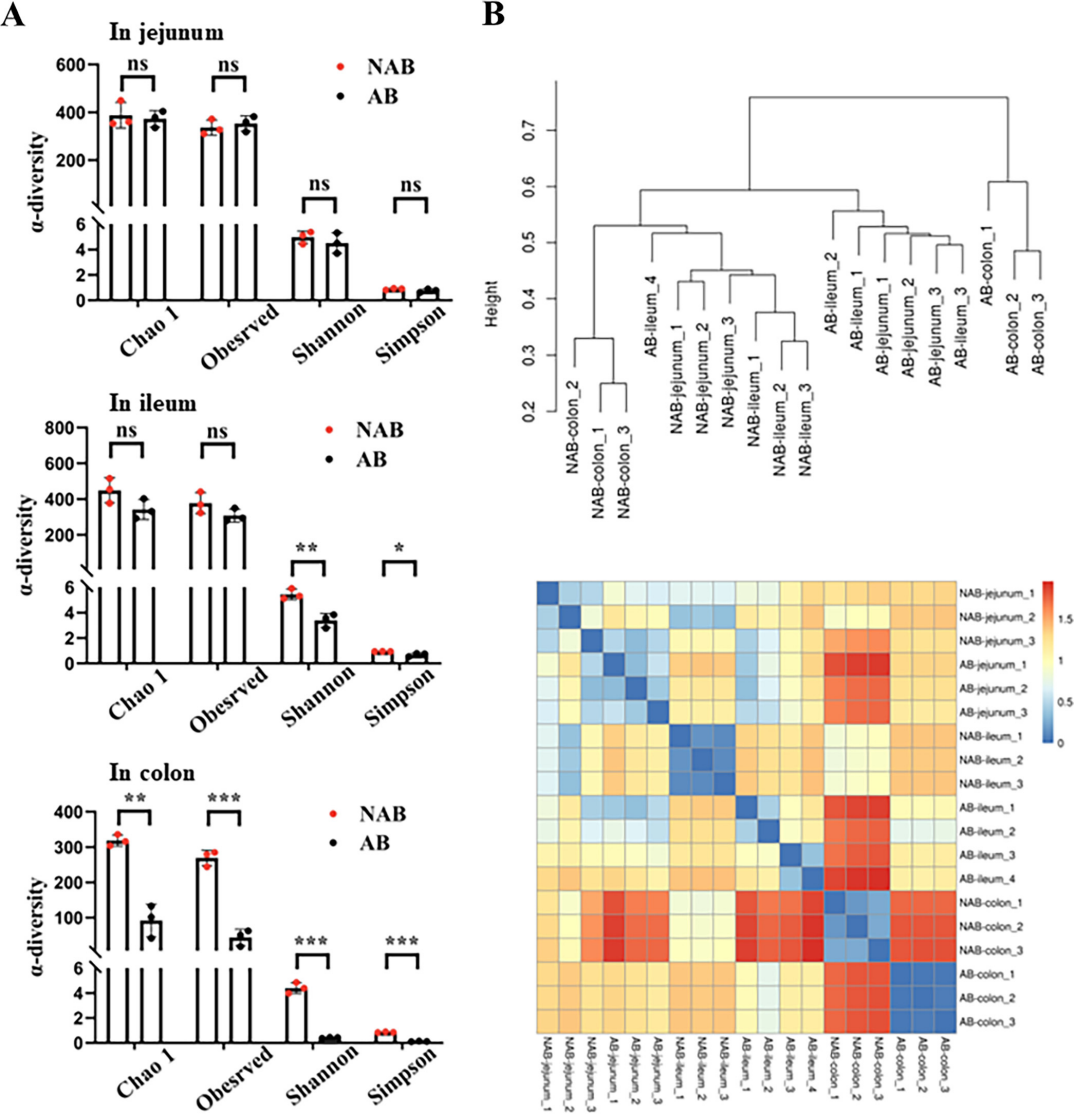
Effect of antibiotics on microbial diversity in mouse jejunum, ileum, and colon. (A) α-Diversity of gut microorganisms, including the Chao index, observed species index, Shannon index, and Simpson index. (B) β-Diversity was expressed using distances containing weighted and unweighted evolution. Data are represented as the mean ± SD (*n* = 3). *, *P* < 0.05; **, *P *< 0.01; ***, *P *< 0.001; ns, not significant.

α-Diversity focuses on the diversity of microbial communities within a sample, including the number, abundance, and homogeneity of species (23). The α-diversity of samples is evaluated using the Chao1 index, observed species index, Shannon index, and Simpson index. The microbial diversity results in the three gut segments are presented in Fig. 2A. The α-diversity of microbiota in the jejunum of mice in the AB group, including the Chao1 index, observed species index, Shannon index, and Simpson index, were not significantly different from those in the NAB group (*P* > 0.05). In the ileum, the Shannon index and Simpson index were significantly lower (*P* < 0.05) in the AB group than in the NAB group. Interestingly, no significant difference (*P* > 0.05) was found in the Chao1 index and observed species index between the AB and NAB groups. The decrease in the Shannon index and Simpson index was weak, though statistically significant. In general, antibiotics did not affect the gut microbiota diversity of the jejunum and ileum.

The Chao1 index is a diversity index that reflects the total number of species by estimating the number of OTUs in the sample. Our assay of colon samples revealed that antibiotics significantly reduced (*P *< 0.01) the Chao1 index, which was consistent with the reduction in colon OTUs in the AB group. The observed species index is used to predict the abundance of microorganisms from the number of OTUs in the measured samples. The observed species index in the AB group was also significantly lower (*P *< 0.001) than that in the NAB group. In addition, the Shannon index and Simpson index are both diversity indices based on the combination of both OTU abundance and OTU uniformity. Antibiotics also significantly reduced the Shannon index and Simpson index in the colon.

The diversity of microorganisms in the small intestine, mainly in the jejunum and colon, is higher than the diversity of microorganisms in the colon (24). This conclusion was verified in this study, where the microbial diversity was higher in the jejunum and ileum of both antibiotic-treated and control mice than in the colon. In addition, the colonic microbial diversity was greatly disturbed by antibiotics. Several studies of flora remodeling in the intestine found that the capacity to receive fecal microbial transplantation (FMT) was much higher in the colon than in the small intestine, and flora remodeling took 8 to 16 days to achieve in the colon, compared with up to 16 to 30 days in the small intestine (12, 25). Another independent study also implied that the colon was more likely to receive exogenous microorganisms to replace its own (26). Overall, our results suggest that antibiotics significantly reduce the diversity of microorganisms in the colon, but not in the small intestine. This provided a novel understanding of FMT realization in the colon.

β-Diversity analysis is a method of comparing the microbial community composition between different groups to obtain the difference in species diversity of samples (23). UniFrac distance analysis (both weighted and unweighted) based on intersample distance is a common route for β-diversity analysis. The results of unweighted UniFrac and weighted UniFrac are presented in Fig. 2B. In brief, the dispersion between the groups of samples was clear, and the intragroup cohesiveness was good. The unweighted UniFrac distance analysis also showed the most significant difference between the colon of mice in the AB and NAB groups.

### Antibiotics depleted the microbial composition of the colon but not that of the jejunum and ileum.

**FIG 3:**
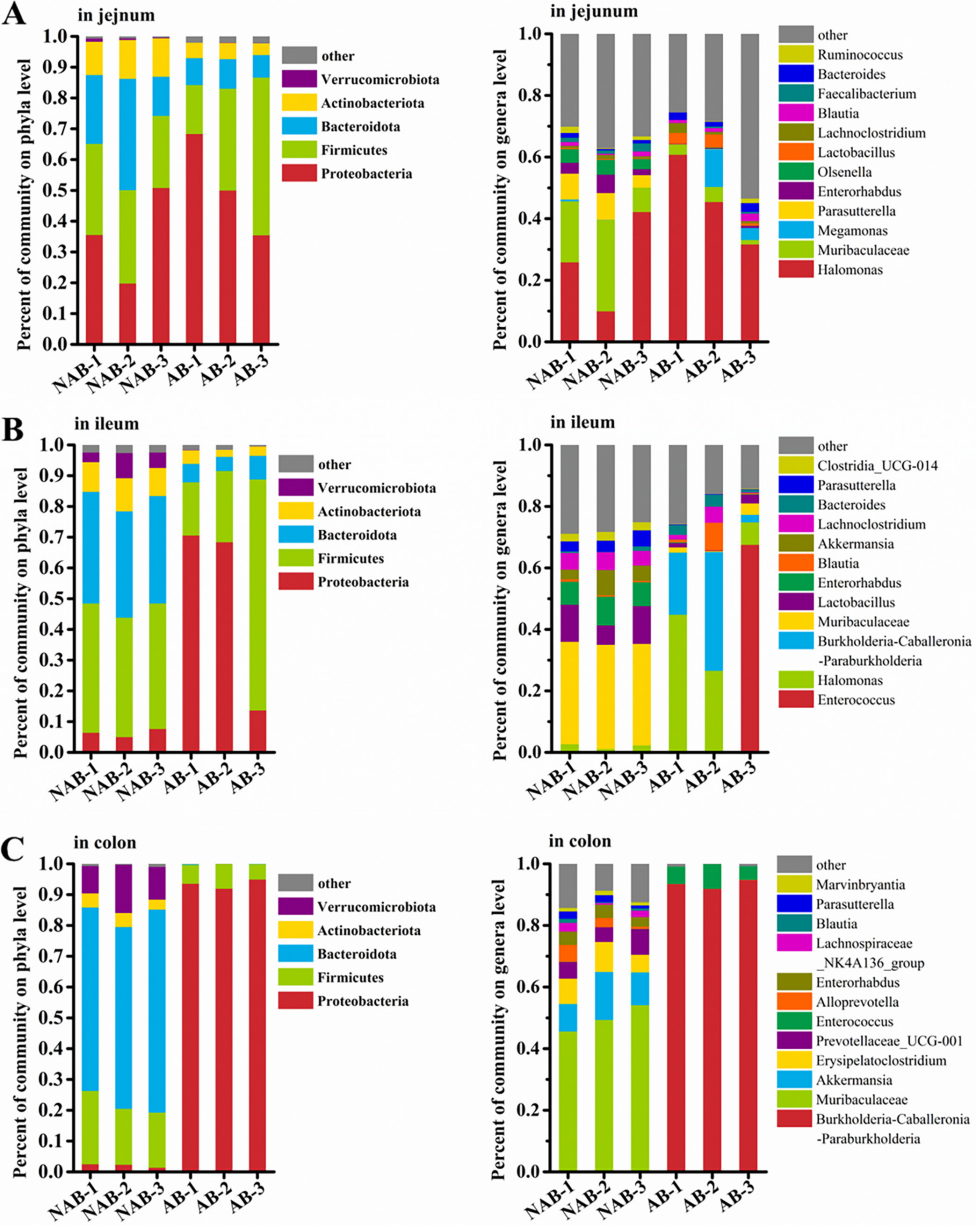
Antibiotics modulate gut microbial composition in mouse jejunum, ileum, and colon. (A and B) The relative abundance of microbes at the phylum level (A) and at the genus level (B).

We analyzed the composition patterns of microorganisms in the jejunum, ileum, and colon at the phylum and genus levels to clarify the site of antibiotic action on the intestine (Fig. 3A to C). In the jejunum, the antibiotic treatment only partially reduced the abundance of *Bacteroidota* and *Actinobacteriota* at the phylum level. This change was small relative to the overall microorganisms of the jejunum at the phylum level. Overall, both AB and NAB group mice had *Proteobacteria*, *Firmicutes*, *Bacteroidota*, and *Actinobacteriota*, close to 98% of all phyla. *Halomonas* and *Muribaculaceae* were the major constituents at the level of all mouse jejunal genera, accounting for half of the jejunal microbial abundance. However, the abundance of *Muribaculaceae* in the AB group was significantly lower than that in the NAB group. In addition, the antibiotic treatment affected the composition of some low-abundance microorganisms. The abundance of *Parasutterella*, *Enterorhabdus*, and *Olsenella* significantly decreased, while that of *Lactobacillus* significantly increased in the AB group compared with the NAB group. These results suggested that antibiotics might affect the microbial composition of the jejunum to some extent. However, this did not achieve the depletion of microorganisms, and only limited alterations affecting the abundance of certain microorganisms existed.

The microbial composition of the ileum is different from that of the jejunum. Compared with *Proteobacteria* as the dominant phylum in the jejunum, the ileum had a higher abundance of *Firmicutes* and *Bacteroidota*, which was up to 75% in the NAB group. However, the perturbation of the ileum by antibiotics seemed to be more dramatic because the AB group still retained a significant percentage of *Proteobacteria*, while the abundance of *Bacteroidota* was greatly reduced. In addition, the microbial composition in the NAB group appeared to be richer, with *Actinobacteriota* and *Verrucomicrobiota* also occupying about 15.3% of the ileal microbial composition, but only about 3.3% was retained after antibiotic treatment. Interestingly, the NAB group presented a more diverse microbial composition at the genus level. The main microorganisms in the NAB group were *Muribaculaceae*, *Lactobacillus*, *Enterorhabdus*, *Akkermansia*, *Lachnoclostridium*, and *Parasutterella*, approaching 70% of the ileal microbial composition. Notably, a decrease in the abundance of all these microorganisms was observed in the AB group. In particular, the decrease in the abundance of *Lactobacillus* and *Akkermansia*, which has a broad beneficial effect in the intestine, was the most pronounced. This might imply that these probiotics in the gut could not tolerate antibiotic interference. The ileal microorganisms in the AB group of mice mainly included *Halomonas* and *Burkholderia-Caballeronia-Paraburkholderia*. A high abundance of *Enterococcus* was also detected in one of the individual mice, while the abundance was low in the other mice, which might be due to individual differences in the degree of antibiotic tolerance. Overall, the ileal microbial results suggested that the pattern of perturbation of the gut microbial composition by antibiotic treatment was drastic. However, this effect might not be aimed at eliminating microorganisms from the gut, as the AB group still retained a fairly rich microbial composition.

Our results suggested that antibiotics had an effect on the composition pattern of microorganisms in the jejunum and ileum. However, the microorganisms were not removed, which implies that the small intestine of mice did not reach a near-sterile state after antibiotic treatment. *Halomonas* was the dominant bacterium in the small intestine, while its abundance was extremely low in the colon. This was consistent with the previously reported results of significant differences in microorganisms in the small and large intestines (27, 28). The natural differences between the small intestine and colon microorganisms may lead to differences in their antibiotic resistance. This in turn explains the ineffectiveness of antibiotics in deleting the action of small intestinal microorganisms while being effective against the colon. The small intestinal flora is an aid to the digestion and absorption of dietary components (3, 29). This is because the small intestinal flora regulates the digestion and absorption of lipids in the intestinal epithelium through mechanisms such as promoting lipase activity in the small intestine and affecting local fatty acid transport (29). Although antibiotics do not completely deplete the small intestinal gut microbes, their effect on the composition pattern of the intestinal microbes cannot be ignored, especially in the ileum. These changes may explain the sudden weight loss in mice after antibiotic treatment. For example, antibiotics reduced the abundance of *Parasutterella*, a small intestinal microorganism associated with the activation of the human fatty acid synthesis pathway (30). Moreover, *Parasutterella* may also be involved in the maintenance of bile acid homeostasis and cholesterol metabolism, thus assisting in the degradation and absorption of dietary fat in the small intestine (31). A reduced abundance of *Parasutterella* is thought to be a mechanism for weight loss (30). In addition, the abundance of some microorganisms generally considered beneficial, such as *Lactobacillus* and *Akkermansia*, is significantly reduced in the ileum. These beneficial microorganisms play an important role in intestinal homeostasis and metabolic function (32, 33).

Subsequently, we analyzed the composition pattern of colon microorganisms. Fortunately, the effect of antibiotics on the removal of colonic microorganisms is tremendous. The NAB group comprised mainly *Bacteroidota*, *Firmicutes*, *Verrucomicrobiota*, *Actinobacteriota*, and *Proteobacteria* with a relative abundance of 61.49%, 19.95%, 8.89%, 4.58%, and 2.46%, respectively. In contrast, the AB group retained only *Proteobacteria* with a relative abundance of 93.45% and *Firmicutes* with an abundance of 6.35%. The 2.46% of *Proteobacteria* before antibiotic treatment became the overwhelmingly dominant microorganisms in the colon after antibiotic treatment, suggesting that microorganisms might have been eliminated in large numbers after antibiotic treatment and that only a small number of antibiotic-tolerant bacteria were retained. The microbial composition at the genus level in the NAB and AB groups once again confirmed this inference. More than 99% of the microorganisms in the AB group were *Burkholderia-Caballeronia-Paraburkholderia* and *Enterococcus*. Furthermore, microorganisms were undetectable at the level of several genera, including *Akkermansia*, *Erysipelatoclostridium*, *Prevotellaceae_UCG-001*, *Alloprevotella*, *Enterorhabdus*, *Lachnospiraceae_NK4A136_group*, *Parasutterella*, and *Marvinbryantia*. These results suggested that the effect of antibiotics on the colon differed from that on the jejunum and ileum. Antibiotics were effective in eliminating the majority of microorganisms from the colon, while only having a limited interfering effect on microorganisms in the jejunum and ileum. This might be explained by the different compositions of anaerobic bacteria in the small intestine and colon. Mowat et al. (34) suggested that aerobic and aerotolerant anaerobic species were prevalent in the small intestine, while strict anaerobes predominated in the colon with low oxygen tension. Clindamycin hydrochloride, the antibiotic selected in this study, primarily targets strict anaerobic species (35). In addition, ampicillin and cefoperazone sodium salt are generally active against Gram-negative and Gram-positive bacteria in the intestinal tract, such as *Enterobacteriaceae* (36, 37).

### Alterations in the levels of intestinal biomarkers induced by antibiotic treatment.

**FIG 4:**
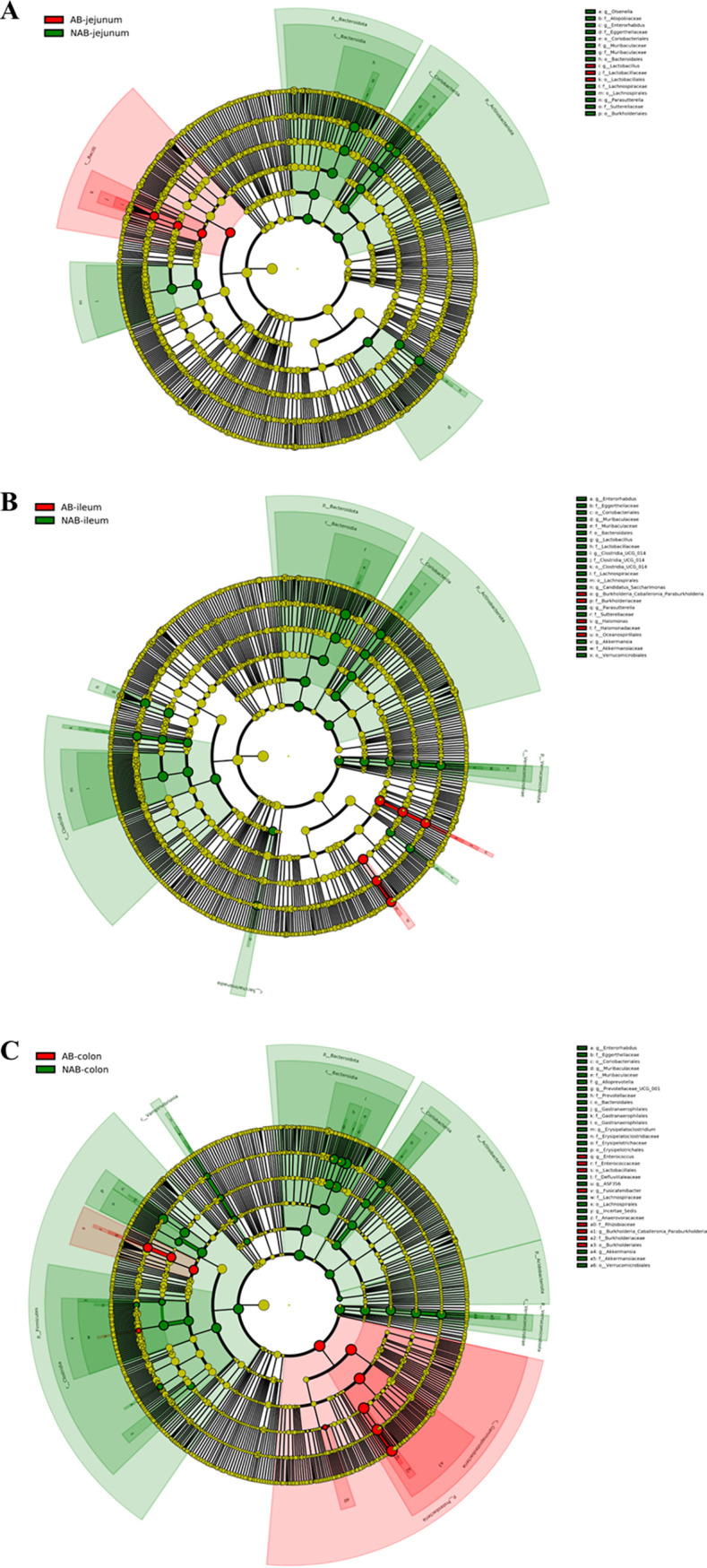
Cladogram maps from LEfSe analysis. (A) jejunum microbiota analysis; (B) ileum microbiota analysis; (C) colonic microbiota analysis. Biomarkers with significant differences follow the group for coloring. Red, AB group; green, NAB group.

**FIG 5:**
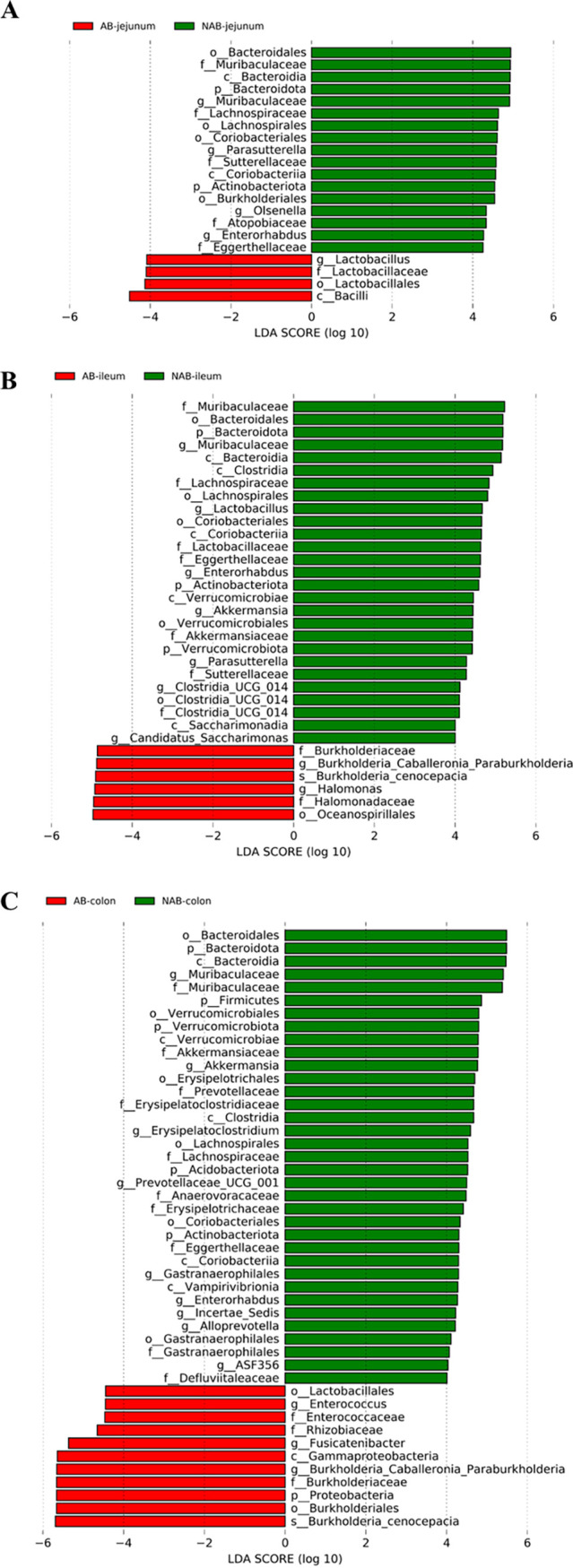
Differential bacterial taxa among different groups meeting LDA values of >4.0 from LEfSe analysis. (A) jejunum microbiota analysis; (B) ileum microbiota analysis; (C) colonic microbiota analysis.

Linear discriminant analysis effect size (LEfSe) is a tool for the discovery and interpretation of high-latitude genomic signature data. It effectively projects the samples in the new space to have the minimum intraclass distance and the maximum interclass distance. This enables comparison between two or more subgroups to find species that differ significantly in abundance between groups, that is, biomarkers (38, 39). The evolutionary branching plots and linear discriminant analysis (LDA) score plots obtained based on LEfSe analysis before and after antibiotic treatment are presented in Fig. 4 and 5. These findings corroborated the results of antibiotic effects on the bacteria at the phylum and genus levels, showing that antibiotic depletion in mice was achieved by acting on the colon.

**FIG 6:**
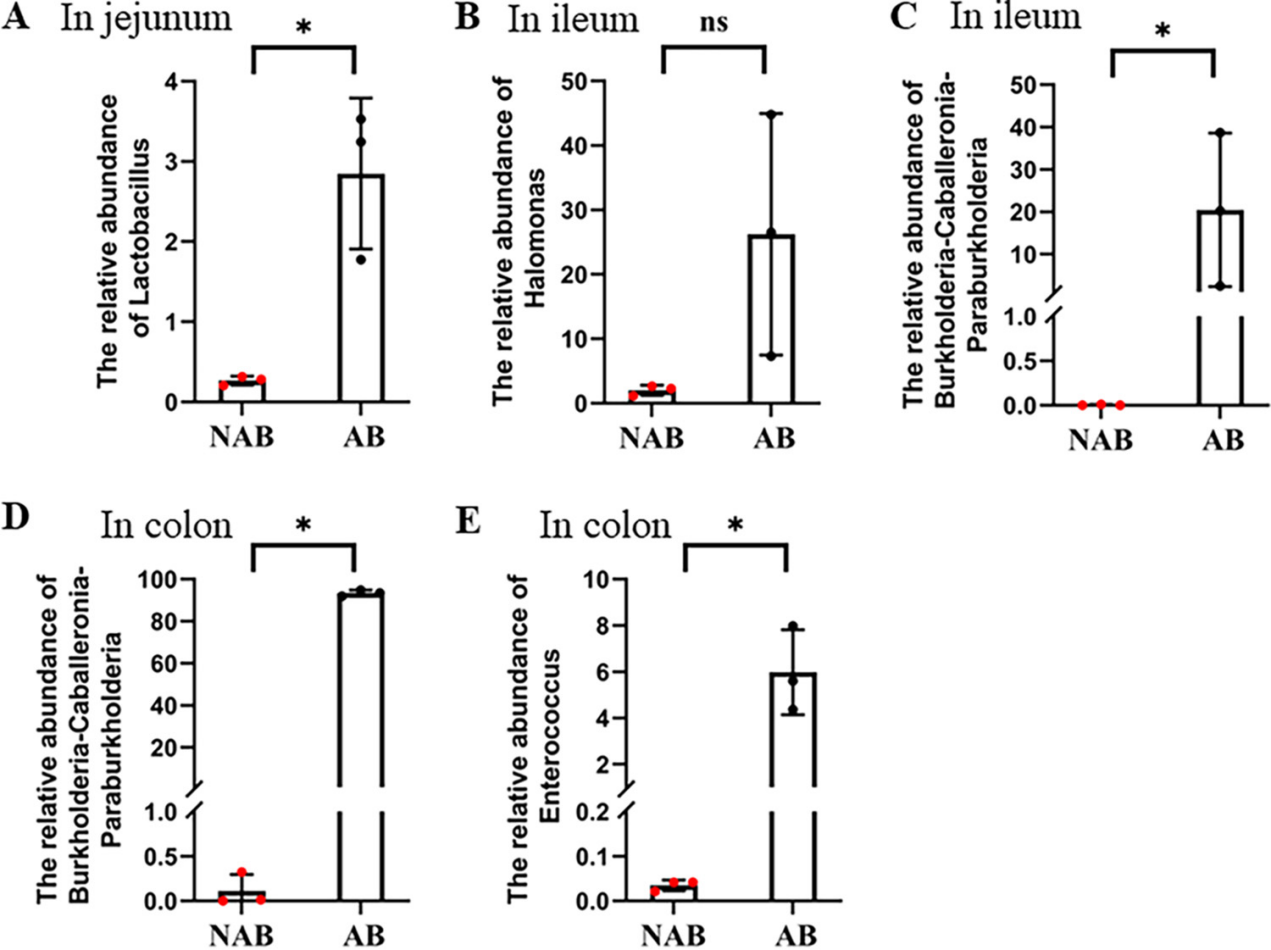
Effect of antibiotic treatment on the abundance of specific microorganisms in the jejunum, ileum, and colon. (A) *Lactobacillus* (in jejunum); (B) *Halomonas* (in ileum); (C) *Burkholderia*-*Caballeronia*-*Paraburkholderia* (in ileum); (D) *Burkholderia*-*Caballeronia*-*Paraburkholderia* (in colon); (E) *Enterococcus* (in colon). Data are represented as the mean ± SD (*n* = 3). *, *P* < 0.05; ns, not significant.

In the jejunum, *Lactobacillus* was screened as a biomarker in the AB group compared with the NAB group, provided that the LDA value of >4 was met. This was consistent with the result at the microbial genus level that the relative abundance of *Lactobacillus* was significantly higher in the AB group (2.85 ± 0.94%) than in the NAB group (0.27 ± 0.06%) (Fig. 6A). In the ileum, the biomarkers in the AB group were *Halomonas* and *Burkholderia-Caballeronia-Paraburkholderia*. These biomarkers reached 46% of the microbial composition in the AB group (Fig. 6B and C). In the colon, *Burkholderia-Caballeronia-Paraburkholderia* and *Enterorhabdus* were screened as the major biomarkers in the AB group. The relative abundance of *Burkholderia-Caballeronia-Paraburkholderia* in the AB group was as high as 93.38 ± 1.44%, while the AB group was almost free of this microorganism, with a relative abundance of only 0.11 ± 1.18% (Fig. 6D). The relative abundance of *Enterorhabdus* was also significantly higher in the AB group (5.89 ± 1.84%) (*P *< 0.01) than in the NAB group (0.03 ± 0.01%) (Fig. 6E). In addition, *Fusicatenibacter* was also considered a biomarker after antibiotic treatment, but its abundance was extremely low in both the NAB and AB groups.

### The microbiome predicted potential functional consequences.

**FIG 7:**
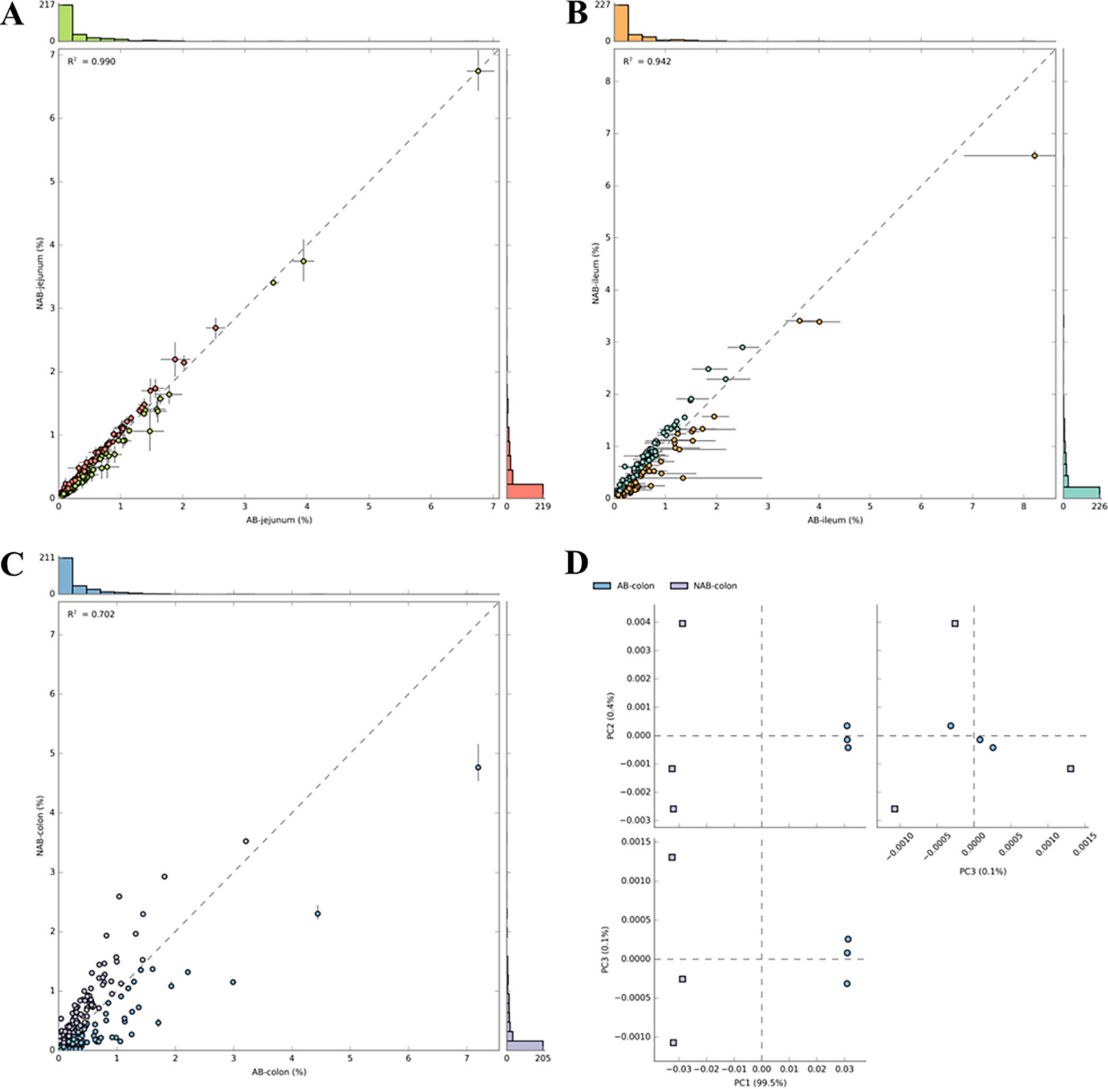
Functional analysis of flora based on gut microbial prediction. (A to C) Scatterplots of the metabolic pathways of (A) jejunum, (B) ileum, and (C) colonic. (D) PCA analysis.

The metabolic pathways of functional genes in the samples were analyzed using Phylogenetic Investigation of Communities by Reconstruction of Unobserved States (PICRUSt), which led to functional differences between samples (40, 41). This prediction was 95% accurate when applied to the analysis of gut microbiota (40). The metabolic pathways predicted for the microbial functions of the jejunum, ileum, and colon were enriched in the scatterplot. The points off the diagonal were considered metabolic pathways affected by antibiotics. In the jejunum and ileum, the predicted metabolic pathways were mainly concentrated on the diagonal (Fig. 7A and B). These results indicated that the functional metabolic pathways predicted based on the jejunum and ileum microbiota were not affected by antibiotics. In the colon, many predicted metabolic pathways were deviated, suggesting that antibiotic treatment significantly affected the underlying metabolic pathways predicted by the microbes (Fig. 7C). This was also confirmed by principal-component analysis (PCA) of colon samples, where the AB group was clearly separated from the NAB group (Fig. 7D).

**FIG 8:**
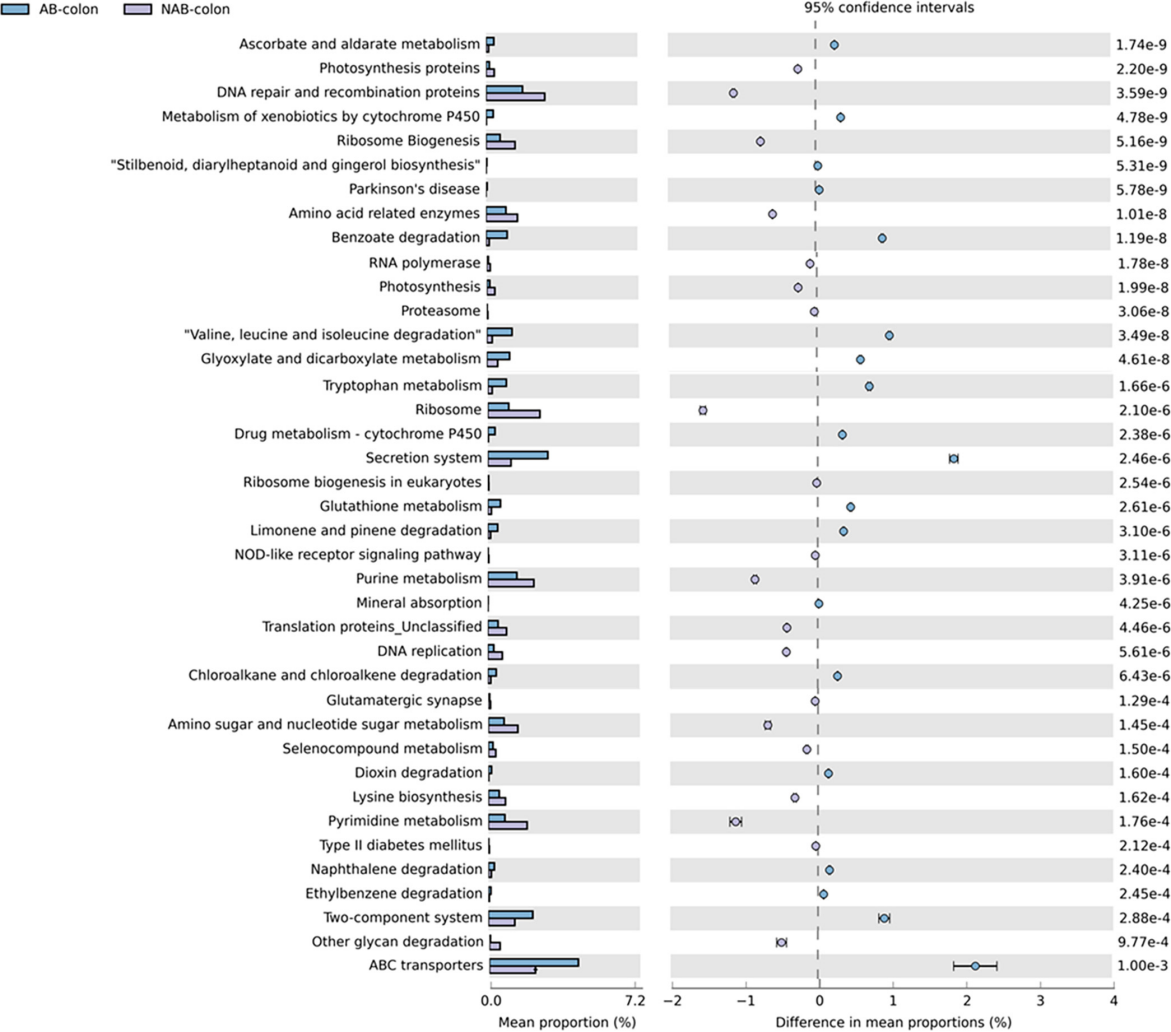
Functional analysis by Tax4Fun of the two groups of colon microorganisms based on Welch’s *t* test. The colored circles represent 95% confidence intervals calculated using Welch’s inverted method.

The differences in metabolic pathways predicted by the colonic microbiota after antibiotic treatment were further analyzed. The results demonstrated that most of the predicted paths in the AB group were significantly different from those in the NAB group (Fig. 8). The predicted metabolic pathways in the AB group that were significantly higher than those in the NAB group were DNA repair and recombination proteins, amino acid-related enzymes, ribosome, biogenesis, purine metabolism, DNA replication, and pyrimidine metabolism. In contrast, the predicted metabolic pathways, including benzoate degradation, fatty acid metabolism, valine, leucine, and isoleucine degradation, the secretion system, ABC transporters, and transporters, were significantly lower in the AB group than in the NAB group.

### Conclusion.

In this study, we used antibiotics to condition the intestinal microbial composition of mice to investigate the effects and loci of antibiotic depletion of intestinal microbes. Interesting results were eventually obtained. After antibiotic conditioning, the mouse colon microbial diversity was significantly reduced, with only a few microorganisms retained at the genus level. The functional predictions showed significant changes from those not conditioned by antibiotics. In contrast, the changes in jejunal and ileal microorganisms were minimal. These results suggested that antibiotics appeared to be effective in removing colonic microbes but not jejunal and ileal microbes. Fixed kinds of antibiotics were a limitation in the present study. Despite the availability of sufficient evidence that these antibiotics are effective, it cannot be ignored that different kinds of antibiotics may have different effects. These elements need more attention in subsequent studies.

## MATERIALS AND METHODS

### Mice.

All mice used were 5-week-old C57BL/6 genotypes and were purchased from the Beijing Vital River Laboratory Animal Technology Co., Ltd. The mice were housed in a suitable living environment with a temperature of 22 ± 2°C and humidity of 45% ± 5%. The living environment alternated between light and dark cycles every 12 h. All mice were randomly divided into cages of 3 each, and they had free access to standard laboratory food and water. Mice were acclimated to feeding for 1 week before entering the antibiotic-treatment study. The mice used in this study were all cared for humanely, and the study was approved by the Northeast Agricultural University Animal Care and Welfare Committee (NEAUEC20210407).

### Antibiotic solution preparation.

The selected antibiotics were based on the “systemic antibiotic cocktail” mixture mentioned in reference 7. This mixed antibiotic solution consists mainly of ampicillin (Beijing Solarbio Science & Technology Co., Ltd., China), cefoperazone sodium salt (Shanghai Yuanye Bio-Technology Co., Ltd, China), and clindamycin hydrochloride (Beijing Solarbio Science & Technology Co., Ltd.). All antibiotics were dissolved in the drinking water in a soluble state and maintained at a concentration of 1 mg/mL of each antibiotic. The drinking water with the antibiotic mixture was changed once a day, and the antibiotics were dissolved in the water the night before and stored at 4°C for backup.

### Antibiotic treatment and sample collection.

The experimental timeline for all mice is shown in Fig. 1A. After 1 week of adaptive feeding, all mice were randomly divided into two groups: the group that received antibiotics (AB group, *n* = 6) and the control group, which did not receive antibiotics (NAB group, *n* = 6). Mice in the AB group received adequate daily drinking water containing a mixture of antibiotics, while the NAB group received plain drinking water. Subsequently, all mice were fasted for 16 h to achieve a fasted state and humanely euthanized with ether. Mouse carcasses were dissected in an ultraclean table, and the contents of the jejunum, ileum, and colon were collected separately. The collected contents were placed in lyophilized tubes and stored promptly in a −80°C freezer until DNA extraction.

### DNA extraction and detection.

DNA was extracted from individual intestinal content samples using the E.Z.N.A. stool DNA isolation kit (Omega Bio-Tek, Norcross, GA, USA). The concentration and purity of the extracted DNA samples were measured using a NanoDrop 2000 spectrophotometer (Shanghai Tucson Vision Technology Co., China), and 1% agarose gel electrophoresis was used to observe the integrity of the DNA samples. Subsequently, the V3 and V4 regions of the 16S rRNA gene were extended using the 338F and 806R primer sets. The specific sequences of 338F and 806R are 5′-ACTCCTACGGGAGGCAGCA-3′ and 5′-GGACTACHVG-GGTATCTAAT-3′, respectively. The thermal program cycle conditions of the amplification process were 95°C for 2 min, followed by the 27-cycle program (at 98°C for 10 s, 62°C for 30 s, and 68°C for 30 s) and a final extension at 68°C for 10 min. The accuracy of the amplified PCR products was tested using 2% agarose gel electrophoresis, and the PCR products were purified using the QuantiFluor-ST purification kit (Biotechnology Co., Ltd., Shanghai, China). Purification, quantification, and sequence PCR were performed using a MiSeq device (Illumina, San Diego, CA, USA) according to the method of reference 42.

### Bioinformatics.

The sequences of 16S rRNA high-variance regions were classified according to the 16S rRNA similarity of microbial populations to obtain the diversity of microbial populations. The initial sequences derived from the raw data were sequence filtered and spliced before analysis. To obtain high-quality clean read, the raw data were further filtered according to the procedure proposed in reference 43. In brief, high-quality sequences were obtained by removing low-quality sequences (length, <220 or >500 nucleotides; average quality score, <20; nitrogenous bases, >3). The paired reads obtained from double-end sequencing were assembled into one sequence based on the principle of overlap relationship to obtain tags with high-variance regions. Then the software FLASH (Fast Length Adjustment of Short reads, v1.2.11) was used to complete the sequence splicing. The sequence splicing conditions were as follows: minimum match length of 15 bp; mismatch rate of 0.1 allowed in the overlapping region. The spliced tags were clustered into operational units (OUTs) using the software USEARCH (v9.2.64), and sequences with >97% homology were grouped into one class of OTUs. Chimeras generated by PCR amplification were removed from the OTU representative sequences using UCHIME (v4.2.40) based on the Chimera database (gold database v20110519).

### Statistical analysis.

The α-diversity of the microbial communities was assessed using the Chao1 index, observed species index, Shannon index, and Simpson index, encompassing measures of microbiota richness and evenness. β-Diversity was expressed using distances containing weighted and unweighted evolution. The UniFrac analysis used evolutionary information between the sequences of each sample to compare whether the samples differed significantly in microbial communities within a particular evolutionary lineage. Nonparametric Kruskal-Wallis rank sums were used to detect species with significant differences in abundance between subgroups. The Wilcoxon rank sum was then used to test the consistency of the differences in species in the previous step across subgroups between groups. Finally, linear regression analysis (LDA) was used to estimate the magnitude of the effect of species abundance on the difference effect for each group. Predicted functional results were obtained by using the Phylogenetic Investigation of Communities by Reconstruction of Unobserved States (PICRUSt) tool to map genus composition information of samples to the genus functional gene composition table of sequenced genomes. The data were analyzed using SPSS v22.0 software (SPSS, Inc., Chicago, IL, USA), and all values were expressed as the mean ± standard deviation (SD). Origin 9 was used for drawing. Values of *P* < 0.05 were considered statistically significant.

### Data availability.

The 16S rRNA sequencing data sets have been uploaded to the Mendeley Data database (https://doi.org/10.17632/j4smf66nsk.1).
